# Impacts of dietary fat on multi tissue gene expression in the desert-adapted cactus mouse

**DOI:** 10.1242/jeb.247978

**Published:** 2024-12-16

**Authors:** Danielle M. Blumstein, Matthew D. MacManes

**Affiliations:** University of New Hampshire, Molecular, Cellular, and Biomedical Sciences Department, Durham, NH 03824, USA

**Keywords:** *Peromyscus*, RNAseq, Dietary fat, Physiology, Multi-tissue

## Abstract

Understanding the relationship between dietary fat and physiological responses is crucial in species adapted to arid environments where water scarcity is common. In this study, we present a comprehensive exploration of gene expression across five tissues (kidney, liver, lung, gastrointestinal tract and hypothalamus) and 17 phenotypic measurements, investigating the effects of dietary fat in the desert-adapted cactus mouse (*Peromyscus eremicus*). We show impacts on immune function, circadian gene regulation and mitochondrial function for mice fed a lower-fat diet compared with mice fed a higher-fat diet. In arid environments with severe water scarcity, even subtle changes in organismal health and water balance can affect physical performance, potentially impacting survival and reproductive success. This study sheds light on the complex interplay between diet, physiological processes and environmental adaptation, providing valuable insights into the multifaceted impacts of dietary choices on organismal well-being and adaptation strategies in arid habitats.

## Teaser

**Summary:** Desert-adapted cactus mice fed a lower-fat diet show altered gene expression relating to mitochondrial function, immune function and circadian rhythm, highlighting the complex interplay between diet, physiological processes and environmental adaptation.

## INTRODUCTION

Harsh environments (e.g. high altitude, deep sea, deserts) represent natural experiments, given that organisms living in these extreme environments have evolved physiological, biochemical and genomic mechanisms to persist (Blumstein and MacManes, 2023; 2024; Blumstein et al., 2024; Kordonowy et al., 2017; Tigano et al., 2022; Wilsterman and Cheviron, 2021). One way for organisms to survive under extreme conditions is for them to carefully manage their intake of food by making strategic decisions based on environmental conditions. Understanding how animals respond opportunistically to diet variation in extreme habitats can provide insight into the adaptive mechanisms they have evolved as well as the potential for other species to adapt to increasingly erratic climatic patterns. As the planet continues to undergo unprecedented climate change, increased desertification and a decrease in free water availability is predicted (IPCC, 2019; Mirzabaev et al., 2019). Studies have demonstrated or predicted distributional changes in extant species in response to changing climate (Moritz et al., 2008; Parmesan, 2006), but fewer have examined the physiological (Bijlsma and Loeschcke, 2005; Blumstein and MacManes, 2023; Blumstein et al., 2024; Gabriel, 2005; Ramirez et al., 2022) and genomic (Blumstein and MacManes, 2024; Cheviron et al., 2014; Colella et al., 2021a; MacManes, 2017; Tigano et al., 2020) mechanisms that may allow animals to adapt in place.

Animals consume three primary macromolecules – carbohydrates, fats and proteins – which exhibit variation in water content (Mellanby, 1942). The oxidation of carbohydrates yields 0.60 g of metabolic water per gram, fats yield 1.07 g per gram, attributed to the higher oxygen demands of lipid metabolism, and proteins yield the least metabolic water (0.41 g) among macronutrients, requiring water loss for the excretion of nitrogenous waste (Davidson and Passmore, 1963; Mellanby, 1942; Schmidt-Nielsen and Adolph, 1964). Energy content also varies significantly, with carbohydrates and proteins providing 16.74 kJ g^−1^, while fats offer more than double the energy at 37.66 kJ g^−1^ (Sánchez-Peña et al., 2017). Further, dietary composition is linked to metabolic water production, generated through the endogenous catabolism of carbohydrates, fats or proteins. In deserts, where water is scarce, both dietary composition and metabolic water production are crucial for survival.

In deserts, the stressors related to the lack of water are often further compounded by extreme temperatures, which increases the rate at which water is lost via evaporation. If the lack of water and extreme temperatures do not require more energy than an organism's ability to absorb from the environment, the organism will maintain energy homeostasis (Romero, 2004). In arid environments such as deserts, the interplay of both preexisting (dietary) water and internally generated water becomes crucial for survival. Food serves as the foundational resource for generating metabolic water but incurs costs during breakdown, leading to water loss through urination and feces production (Schmidt-Nielsen, 1975). In desert ecosystems, rodents primarily subsist on vegetation, seeds and insects, each exhibiting variation in fat, protein and carbohydrate content (Orr et al., 2015; Wolf and del Rio, 2003). However, the specific composition of the diet is often unpredictable and highly diverse, especially for wildlife. Factors such as energy potential, nutritional content and the capacity for water production (Schmidt-Nielsen and Adolph, 1964) are all contingent on the composition of the diet.

Faced with the natural variation in resource availability, opportunistic animals such as the desert-adapted cactus mouse (*Peromyscus eremicus*) have the capacity to significantly impact their internal water balance and, in turn, their survival. *Peromyscus eremicus* are not skilled burrowers (Hu and Hoekstra, 2017; Weber and Hoekstra, 2009), limiting the effects of a microclimate effect; however, they are omnivorous, utilizing seeds, arthropods and green vegetation seasonally (Bradley and Mauer, 1973; Meserve, 1976), and shift to consuming cactus seeds and fruit pulp during summer months (Orr et al., 2015). When comparing a standard diet with a low-fat diet, Blumstein et al. (2024) described elevated rates of water loss during the warmer, drier light phase compared with the dark phase. Variations in serum electrolyte levels suggest that dietary fat plays a significant role in regulating water balance during long-term physiological experiments. Even minor variation in water balance can profoundly affect cognitive function (Riebl and Davy, 2013), physical performance and, ultimately, survival and reproductive success (Cunningham et al., 2021).

Metabolic flexibility is dependent on the capacity of reciprocal regulation of glucose use and fatty acid oxidation in the mitochondria (Muoio, 2014). When glucose consumption increases, fatty acid oxidation is suppressed, and vice versa (Hue and Taegtmeyer, 2009). During the postprandial phase following a carbohydrate-rich meal, glucose uptake, glycolysis and pyruvate oxidation are favored because of the elevated glucose and insulin levels (Muoio, 2014). Consequently, fatty acids are directed towards triglyceride synthesis and storage. Moreover, the availability of fat inhibits glycolysis, hindering glucose uptake and utilization (Randle et al., 1963).

During water deprivation, animals have been observed to reduce solid food intake, a phenomenon known as dehydration-associated anorexia, which minimizes water needed for digestion and allows for water reabsorption from the kidneys and gastrointestinal (GI) tract (Armstrong et al., 1980; Blumstein and MacManes, 2024; Hamilton and Flaherty, 1973; Rowland, 2007; Salter and Watts, 2003; Schoorlemmer and Evered, 2002; Watts and Boyle, 2010). Glucose levels are maintained during limited food intake and water deprivation as a result of enhanced glycogenolysis and gluconeogenesis (Blumstein and MacManes, 2024; Salter and Watts, 2003; Schoorlemmer and Evered, 2002; Watts and Boyle, 2010). The inhibition of glycolysis impedes glucose metabolism (Spriet, 2014). This regulatory mechanism creates a positive feedback loop that promotes fatty acid oxidation during nutrient scarcity, preserving glucose for essential biosynthetic processes and brain metabolism.

Food intake and composition also serve a twofold function, acting as both a source of energy and a regulator of the GI tract (Henderson et al., 2015; Keeney and Finlay, 2011; Wu et al., 2011). Nutrients, obtained from the host, play a crucial role in shaping the composition of host-associated microbial communities (McFall-Ngai, 2007). Despite the mutually beneficial relationship between intestinal microbes and the host, the proximity of an abundant bacterial community to intestinal tissues poses significant health risks. Thus, the gut microbiota play a crucial role in diet processing but, simultaneously, dietary changes can influence the composition of the gut microbial community (Sonnenburg et al., 2016).

To fully understand the mechanisms that control organismal health, responses must be studied in parallel and at multiple levels. Here, we extend previously characterized metabolic patterns for male and female cactus mice fed an experimental diet low in fat to those on a standard laboratory diet (Blumstein et al., 2024) with a thorough examination of gene expression patterns in five tissues known to be involved in water homeostasis. The liver was targeted as a primary site of metabolism and therefore water production. The kidney was included as it is the primary source of osmoregulation, controlled by hormones produced in the hypothalamus. Gene expression in the lung was assayed as a substantial amount of water is lost via respiration. In our previous study (Blumstein et al., 2024) we showed a distinct relationship between water loss rate and diet composition. Expression changes observed in the lung may serve to limit water loss and could have a large effect on total body water balance. Lastly, the GI tract was investigated because of its involvement in diet processing and dehydration anorexia.

We describe the whole-organism physiological and genomic response to variations in dietary fat in desert-adapted opportunistic animals. Overall, the GI tract experienced the largest number of changes in gene expression, followed by the lung, hypothalamus, kidney and liver. A network-based statistical approach that identifies clusters of genes with highly correlated expression profiles (modules), a weighted gene correlation network analysis (WGCNA; Langfelder and Horvath, 2008), of the kidney, liver and lung revealed significant modules related to circadian rhythm while the hypothalamus, kidney, liver and lung all had significant differentially expressed genes related to mitochondrial gene expression. Additionally, we highlight the significance of dietary fat in the immune response. These findings suggest that a low-fat diet may limit the survival of the cactus mouse in the event of restricted access to water, a common challenge in arid habits.

## MATERIALS AND METHODS

### Animal care and RNA extraction

Captive-bred, sexually mature, non-reproductive healthy male and female *Peromyscus eremicus* (Baird 1858) raised in an environmental chamber replicating the climate of the Sonoran Desert were used in this study (Blumstein and MacManes, 2023; Blumstein et al., 2024; Colella et al., 2021b; Kordonowy et al., 2017). Within the chamber, the light phase lasted 12 h (08:00 h to 20:00 h) at 32°C and 10% relative humidity (RH), followed by a 1 h transition to 24°C and 25% RH for the 9 h dark phase (21:00 h to 06:00 h). The room cycle concluded with a 2 h transition back to the light phase conditions. We adhered to animal care procedures established by the American Society of Mammologists and sanctioned by the University of New Hampshire Institutional Animal Care and Use Committee (protocol number 210604). All mice underwent routine animal care, including health evaluations conducted by licensed veterinary professionals, prior to the experiment. No health concerns were identified during these assessments and none of the animals were removed prematurely. All mice remained active for the duration of the experiment.

The mice (*n*=28 males, *n*=28 females), provided with water *ad libitum*, were randomly assigned to one of two diet treatment groups, a standard diet group [SD – LabDiet^®^ 5015*: 26.101% fat, 19.752% protein, 54.148% carbohydrate, energy 15.02 kJ g^−1^, food quotient (FQ, the theoretical respiratory quotient produced by the diet based on macronutrient composition; Westerterp, 1993) 0.89] or a low-fat diet group [LF diet – Modified LabDiet^®^ 5015 (5G0Z), 6.6% fat, 22.8% protein, 70.6% carbohydrate, energy 14.31 kJ g^−1^, FQ=0.92].

Prior to the beginning of the experiment, mice were fed the assigned diet for 4 weeks. We weighed and transferred each mouse to an experimental chamber 24 h prior to the beginning of metabolic measurements. After the 24 h chamber acclimation period, we collected 72 h of undisturbed metabolic data. Each chamber was provided with water and the assigned diet *ad libitum*. Throughout the study, a pull flow-through respirometry system (Lighton, 2018) was used to assess metabolic phenotypes, computing CO_2_ production rates (*V̇*_CO_2__), O_2_ consumption (*V̇*_O_2__) and water loss rate (WLR) using equations 10.6, 10.5 and 10.9, respectively, from Lighton (2018). Respiratory quotient (RQ, the ratio of *V̇*_CO_2_ _to *V̇*_O_2__) and energy expenditure (EE; kJ h^−1^) were calculated as in equation 9.15 of Lighton (2018). For downstream analysis, we calculated the mean water loss, EE and RQ of the last hour for each mouse. At the conclusion of the experiment (day 3), weight was again recorded, the mice were euthanized with an overdose of isoflurane, and trunk blood was collected. Using an Abaxis i-STAT^®^ Alinity machine and CHEM8+ cartridges (Abbott Point of Care Inc., Abbott Park, IL, USA) we measured the concentration of sodium (Na), potassium (K), chloride (Cl), creatinine (Cr), blood urea nitrogen (BUN), hemoglobin (Hb), hematocrit (Hct), glucose (Glu), anion gap (AnGap), total CO_2_ (*T*_CO_2__) and ionized calcium (iCa), all of which are expected to vary in response to hydration status and renal function. Finally, using sodium, glucose and BUN, we calculated osmolality using the formula in Rasouli (2016).

Following similar methods to Blumstein and MacManes (2024), the lung, liver, kidney, a section of the GI tract and hypothalamus were collected, preserved, and RNA was extracted from anatomically similar regions of the tissue using a standardized Trizol protocol. RNAseq libraries were prepped for sequencing using a poly-A tail purification, barcoded using Illumina primers, and dual-barcoded using a New England Biolabs Ultra II Directional kit (NEB #E7765). Individually labeled samples were pooled and sequenced using a paired-end protocol and a read length of 150 bp, across two lanes of a Novaseq 6000 at the University of New Hampshire Hubbard Center for Genome Studies.

### Computational analysis

Demultiplexed reads were aligned to the *P. eremicus* genome version 2.0.1 from the DNA Zoo Consortium (dnazoo.org) using STAR v.2.7.10b (Dobin et al., 2013) allowing a 10 base mismatch, a maximum of 20 multiple alignments per read, and discarding reads mapped at <30% of the read length. Aligned reads were counted using HTSEQ-COUNT v.2.0.2 (Anders et al., 2015) with the flags ‘-s no -t exon’. Count files were imported into R v.4.0.3 (http://www.R-project.org/). Counts were merged into a gene-level count by combining all counts that mapped to the same gene. Low expression genes were removed if the gene had 10 or fewer counts in 8 or more individuals across the experiment.

Differential gene expression analysis was done using DESEQ2 (Love et al., 2014) on the dataset as a whole with three different models that tested for the effects of sex, diet and tissue type using an LRT test (∼trt+tissue, ∼trt+sex, ∼sex+tissue). Downstream analysis using the dataset as a whole was done using the results from this model: ∼sex+tissue+trt, test=‘LRT’, ∼sex+tissue. We also analyzed each tissue independently with two different models (∼trt and ∼sex), testing the effect of and identifying genes specific to sex and diet with a Wald test (contrast=standard, low fat and contrast=M, F). We used a Benjamini and Hochberg (1995) correction for multiple comparisons and an alpha value of 0.05 for significance. To better link within-tissue patterns of gene expression with treatment, condition or collected phenotypic data, we conducted an analysis in WGCNA (Langfelder and Horvath, 2008) for each tissue independently and included the physiological phenotypes collected [mean EE, WLR and RQ, mass, sex, diet, AnGap, *T*_CO_2__ and the panel of electrolytes (Na, K, Cl, Cr, BUN, Hb, Hct, Glu, iCa and osmolality, calculated from sodium, glucose and BUN)]. Canonical correlation analysis (CCA) in the R package vegan (http://vegan.r-forge.r-project.org/) was performed to explore gene expression correlations across tissues, diet, sex and metabolic variables. ANOVA was used to identify what response variables were significant for graphing on the triplot, and allowed us to visualize their correlative nature; vectors pointing in the same direction are positively correlated, while vectors pointing in opposite direction are negatively correlated. To identify genes of interest, we selected genes that graphed two standard deviations away from the origin for CCA1 and CCA2. Lastly, gene ontology (GO) analyses were conducted using the R package gprofiler (Kolberg et al., 2023) using the default parameters, and identified the top-most 20 significant GO terms based on g:SCS corrected *P*-values (Reimand et al., 2007) to elucidate significant gene functions and pathways associated for each upregulated and downregulated list for each tissue and for each significant module from the individual WGCNA analysis. The full analytical code and data are available from the GitHub repository (https://github.com/DaniBlumstein/diet_rnaseq). Finally, we compiled a list of genes with high confidence from the findings of two of the independent analyses: WGCNA and genes located two standard deviations from the origin in the CCA.

## RESULTS

### Genomic data

On average, we generated 13.76 million reads per sample, with a standard deviation of 35.08 million reads. On average, 78.15% of reads per sample were uniquely mapped, with a standard deviation of 0.03%. Detailed information regarding the number of reads and per-sample mapping rates can be found in Table S1. Raw read files are archived at NCBI SRA BioProject: PRJNA1048512, and the gene expression count data along with the analytical code are accessible in the GitHub repository (https://github.com/DaniBlumstein/diet_rnaseq).

### Electrolytes and physiological phenotypes

The same mice used to generate the electrolyte data and to describe the cyclical pattern of the physiological data collected throughout the 72 h data collection in Blumstein et al. (2024) were also used in the study described herein for a snapshot RNAseq analysis. There were no significant differences in mass between males and females [mean of 22.2 g (range: 15.40–29.10 g) versus 21.2 g (range: 16.91–29.34 g), respectively; Table 1]; however, when comparing diet groups, females on the SD weighed significantly more than females on the LF diet (*P*=0.03). When comparing males and females separately, the following blood measurements were significantly different for the SD versus the LF diet treatments: sodium *P*_f_=0.0075 (elevated in LF); potassium *P*_m_=0.028 (elevated in SD) and *P*_f_=0.0099 (elevated in SD); BUN *P*_m_=0.027 (elevated in LF); Hct *P*_m_=0.04 (elevated in LF) and *P*_f_=0.02 (elevated in LF); iCa *P*_m_=0.0059 (elevated in LF) and *P*_f_=0.00059 (elevated in LF); glucose *P*_f_=0.011 (elevated in LF); osmolality *P*_m_=0.056 (elevated in LF) and *P*_f_=0.003 (elevated in LF); *T*_CO_2__ *P*_m_=0.002 (elevated in LF); AnGap *P*_f_=0.024 (elevated in LF) and *P*_m_=0.017 (elevated in LF) (Table 1). When comparing males and females on the same diet, the following electrolytes were significantly different: sodium (*P*_SD_=0.028, elevated in males) and osmolality (*P*_SD_=0.039, elevated in males) differed significantly for mice on the SD (Table 1). No electrolyte measurements differed between males and females on the LF diet (Table 1).

**Table 1.**
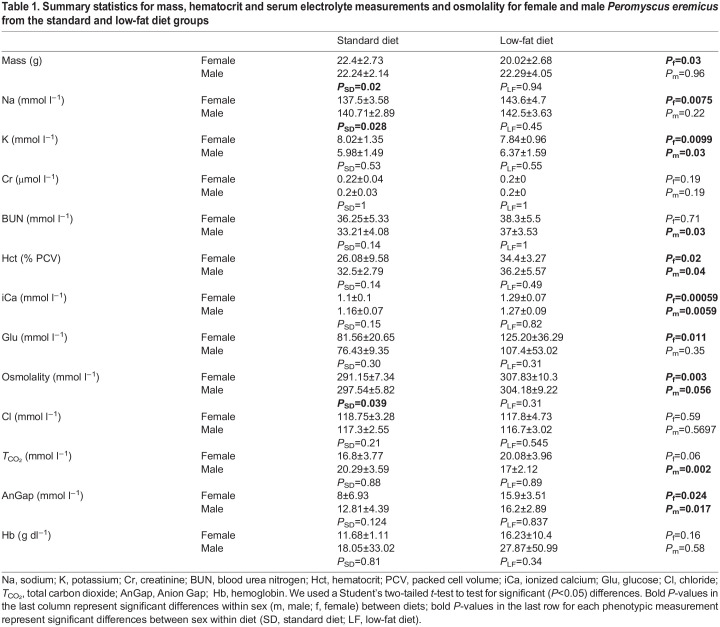
Summary statistics for mass, hematocrit and serum electrolyte measurements and osmolality for female and male *Peromyscus eremicus* from the standard and low-fat diet groups

Using the data collected in Blumstein et al. (2024) (see https://github.com/DaniBlumstein/Diet_paper), we calculated the means for EE, WLR and RQ for the same 28 adult females and 28 adult males (*n*=14 of each treatment, total *n*=56) for each mouse from the last hour of data collection. Within sex, EE significantly differed between diets for males (*P*=0.012) but not females (*P*=0.167); WLR was significantly different between diets for males (*P*=0.001) and females (*P*=2.641e−05); RQ was not significantly different.

### Differential gene expression

Following the methods in Blumstein and MacManes (2024), we cross-referenced our gene IDs with *Homo sapiens* gene IDs and filtered low-expression genes, leaving 17,012 genes in the dataset as a whole. Patterns of gene expression data are largely driven by tissue type (PC1: 44% variance and PC2: 23% variance; Fig. S1).

**Fig. 1.**
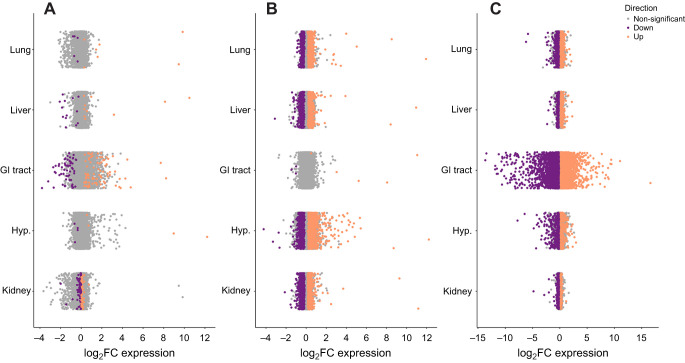
**Differential gene expression of *Peromyscus eremicus* fed a standard or low-fat diet.** Data are log fold-change of expression of all genes across the lung, liver, gastrointestinal (GI) tract, hypothalamus (Hyp.) and kidney, comparing males with females on the standard diet (A), males with females on the low-fat diet (B) and all mice on the standard diet with all mice on the low-fat diet (C). Purple (downregulated) and orange (upregulated) colored dots indicate *P*<0.05, whereas gray dots indicate *P*≥0.05.

All downstream analysis, with the exception of CCA (see below) was done one tissue at a time by filtering the count and sample data files by the tissue of interest for that analysis. This left 12,935 genes in the lung, 10,820 genes in the liver, 12,249 genes in the GI tract, 13,037 genes in the hypothalamus and 11,950 genes in the kidney. Each tissue had a unique number of differentially expressed genes (*P*<0.05) between diet treatment and sex (Fig. 1, Table 2).

**Table 2.**
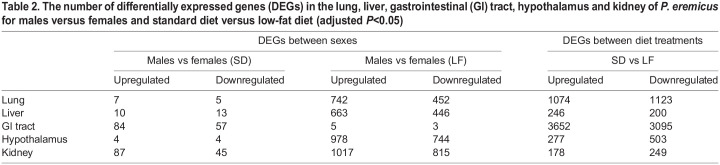
The number of differentially expressed genes (DEGs) in the lung, liver, gastrointestinal (GI) tract, hypothalamus and kidney of *P. eremicus* for males versus females and standard diet versus low-fat diet (adjusted *P*<0.05)

### Weighted gene correlation network analysis

**Fig. 2.**
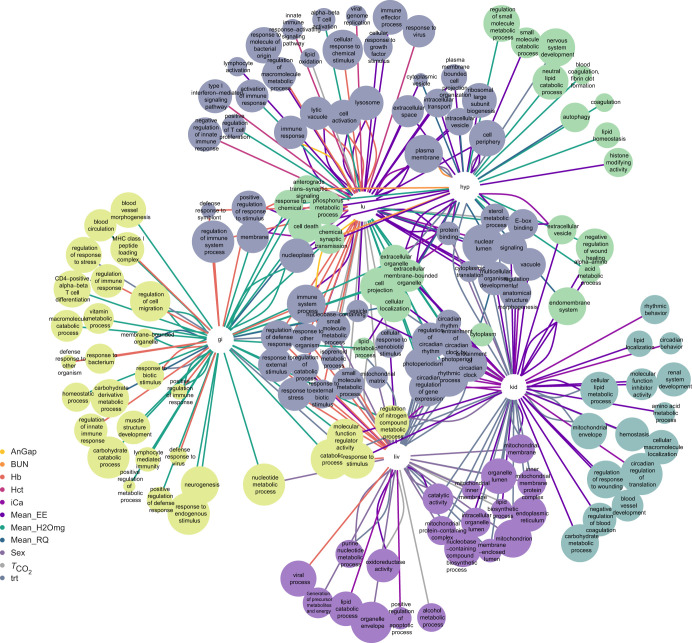
**Visualization of a subset of gene ontology (GO) terms to show common significant weighted gene correlation network analysis (WGCNA) modules within and between the tissues of *P. eremicus*.** Selections of the top 20 significant GO terms for each significant phenotype module combination are shown. The number of genes in the GO term is indicated by the size of the node and the color represents the phenotype for that module. Clusters are placed according to the Kamada–Kawai algorithm (Kamada and Kawai, 1989) and are largely driven by tissue followed by shared GO terms between tissues. A summary of the GO terms shared across tissues and/or significant modules can be found in Tables 3 and 4 while the full list of GO terms by tissue can be found in Table S2. AnGap, anion gap; BUN, blood urea nitrogen; Hb, hemoglobin; Hct, hematocrit; iCa, ionized calcium; mean_EE, mean energy expenditure; mean_H2Omg, mean water loss; mean_RQ, mean respiratory quotient; *T*_CO_2__, total CO_2_; trt, diet treatment.

Briefly, WGCNA is a network-based statistical approach that identifies clusters of genes with highly correlated expression profiles (modules), or genes that could function together in a pathway (Langfelder and Horvath, 2008). For the lung, we identified 30 modules, of which 23 were significant, with each module containing 20–8292 genes (Fig. 2). Notably, nine modules were significant for three or more phenotypes. In the liver, 40 modules were identified, 26 of which were significant and contained 20–14,458 genes per module. Seven of these modules were significant for three or more phenotypes. In the GI tract, 32 modules were identified, and 25 were significant, each with 21–7599 genes. Fifteen modules were significant for three or more phenotypes. In the hypothalamus, we found 21 modules, of which 18 were significant, and they contained 23–9472 genes per module. Ten modules were significant for three or more phenotypes. Finally, in the kidney, we identified 37 modules, 25 of which were significant and contained 21–13,762 genes per module. Seven of these modules were significant for three or more phenotypes. Several phenotypes (Na, Bun, AnGap, K, *T*_CO_2__, iCa, diet treatment and EE) had at least one significant module in all five tissues. There was at least one significant module for sex in the hypothalamus, liver, kidney and lung, and at least one significant module for Hb in the hypothalamus, liver, GI tract and lung. At least one significant module for RQ was in the hypothalamus, liver, GI tract and lung. At least one significant module for Cl was in the hypothalamus, kidney, GI tract and lung. At least one significant module for Hct was in the liver, GI tract and lung. At least one significant module for glucose was in the kidney, GI tract and lung. At least one significant module for WLR was in the hypothalamus, GI tract and lung. At least one significant module for mass was in the liver and kidney and there were no significant modules for creatin in any of the tissues.

**Table 3.**
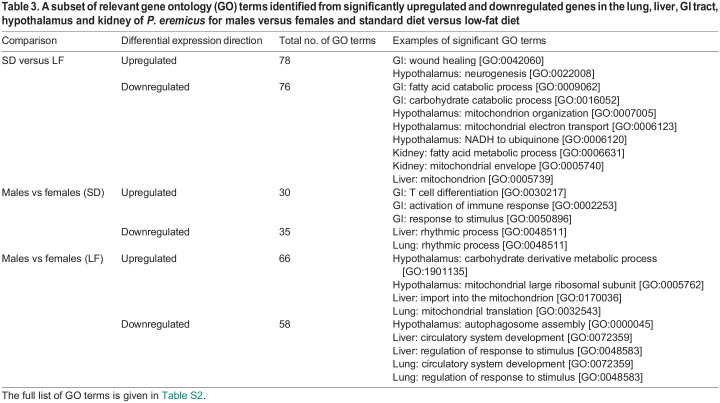
A subset of relevant gene ontology (GO) terms identified from significantly upregulated and downregulated genes in the lung, liver, GI tract, hypothalamus and kidney of *P. eremicus* for males versus females and standard diet versus low-fat diet

**Table 4.**
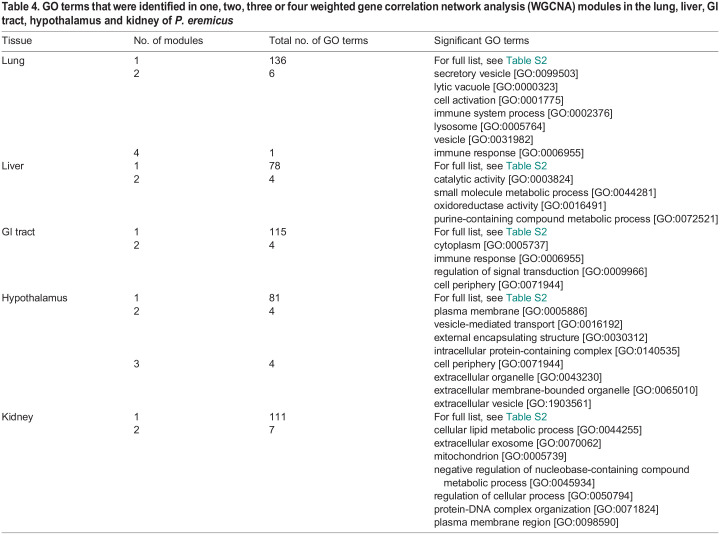
GO terms that were identified in one, two, three or four weighted gene correlation network analysis (WGCNA) modules in the lung, liver, GI tract, hypothalamus and kidney of *P. eremicus*

### Gene ontology

To enable functional interpretation, we filtered to the top 20 GO terms, based on *P*-values, for the upregulated and downregulated significantly differentially expressed genes for each tissue. We observed a total of 343 unique GO terms. Of these, 154 GO terms were found in the diet comparison and 189 GO terms were found in the sex comparison (Table 3). GO analysis of the genes contained in significant WGCNA modules identified a total of 455 unique GO terms. Specifically, in the lung, 143 unique GO terms were identified, in the liver, 82 unique GO terms were found, for the GI tract, 119 unique GO terms were identified, in the hypothalamus, 89 unique GO terms were found and, lastly, in the kidney, 118 unique GO terms were identified (Table 4). When comparing GO terms identified in WGCNA modules across tissues, 23 GO terms were for three tissues, six GO terms were for four tissues, and two terms were identified in all five tissues (Table 5). The full list of GO terms is shown in Table S2.

**Table 5.**
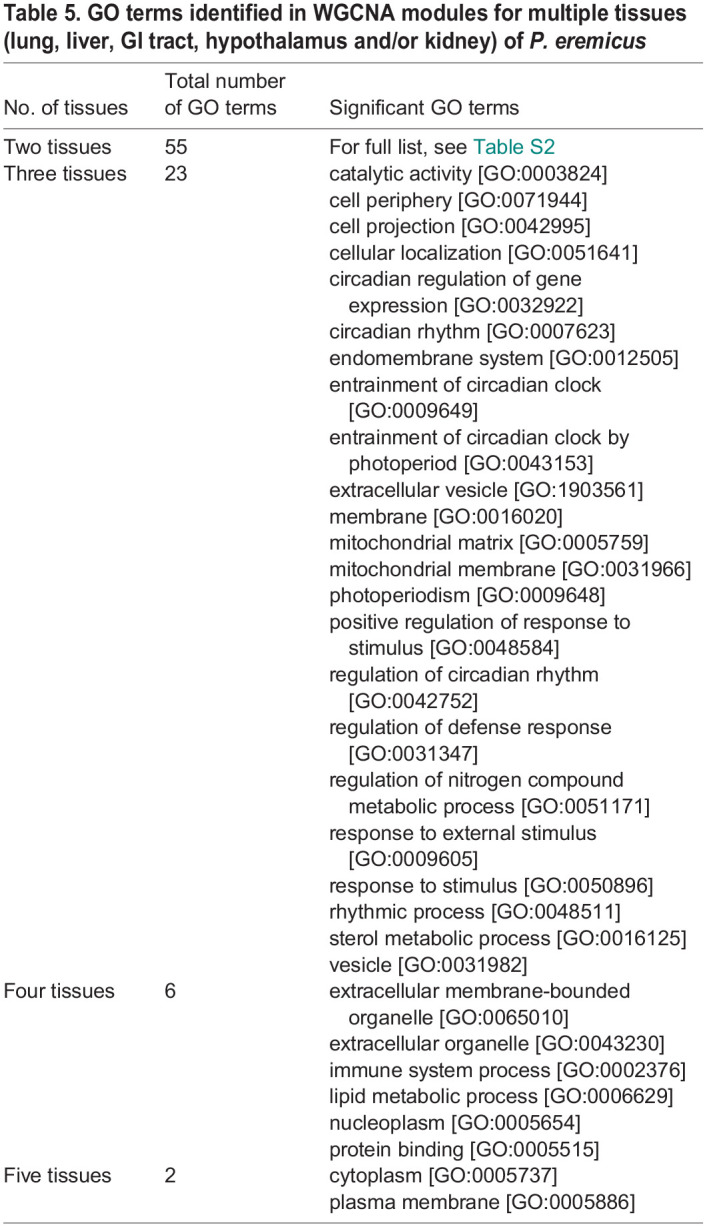
GO terms identified in WGCNA modules for multiple tissues (lung, liver, GI tract, hypothalamus and/or kidney) of *P. eremicus*

### Canonical correlation analysis

**Fig. 3.**
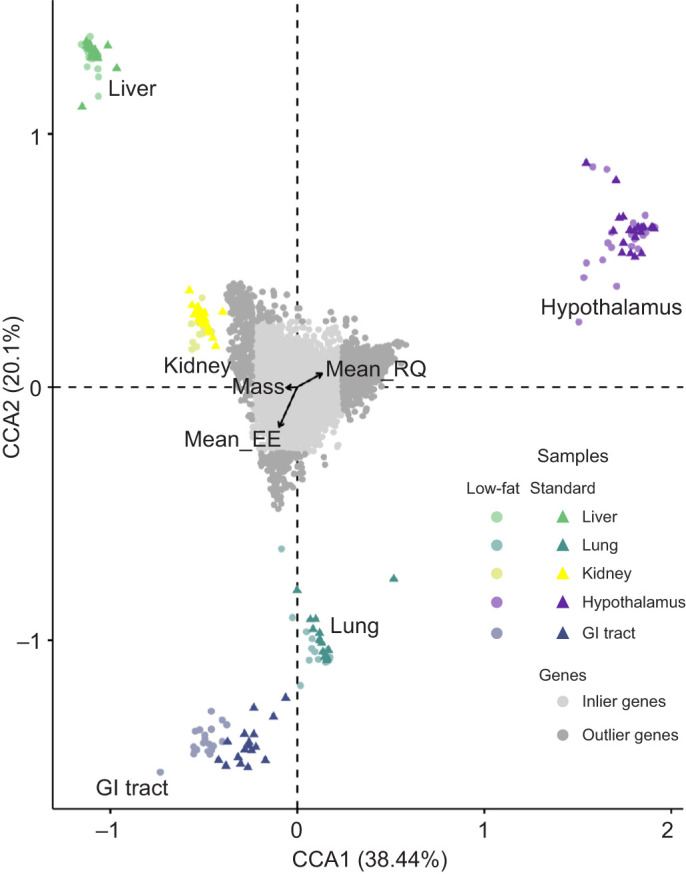
**Canonical correspondence analysis (CCA) indicates correlations between normalized differentially expressed genes and physiological measurements for *P. eremicus* fed a standard diet or a low-fat diet.** The distribution of tissue samples in Euclidian space as a function of their gene expression values is shown (points colored by tissue type and shaped by diet treatment). Outlier genes (defined as two standard deviations or more from the mean) are colored dark gray. Inlier genes (defined as less than two standard deviations from the mean) are colored light gray. CCA reveals a significant relationship between mass (*F*=2.44, *P*=0.046), RQ (*F*=4.02, *P*=0.004) and EE (*F*=3.57, *P*=0.004).

We examined the relationship between gene expression and water access, physiological variables (EE, RQ, WLR and mass), tissue type and sex using CCA (http://vegan.r-forge.r-project.org/). CCA explained a large amount of variance (CCA1: 38.07% and CCA2: 20.09%; Fig. 3). Additionally, 1296 genes were identified as being two standard deviations from the origin. Overall, the model was significant (*P*=0.001; Table 6), meaning that there is an association among our variables. Mass was significant (*F*=2.44, *P*=0.046; Table 6), with the vector pointing at the kidney, RQ was significant (*F*=4.02, *P*=0.004; Table 6), with the vector pointing at the hypothalamus, and EE was significant (*F*=3.57, *P*=0.004; Table 6) with the vector pointing at the GI tract. This suggests that gene expression in the hypothalamus, kidney and GI tract affects RQ, mass and EE differently.

**Table 6.**
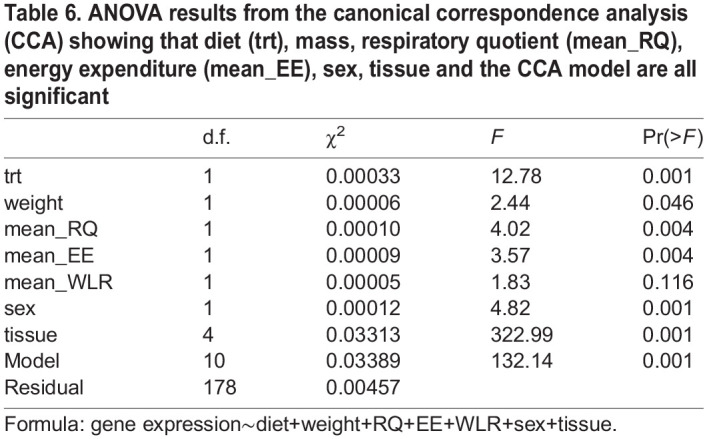
ANOVA results from the canonical correspondence analysis (CCA) showing that diet (trt), mass, respiratory quotient (mean_RQ), energy expenditure (mean_EE), sex, tissue and the CCA model are all significant

### Consensus gene set

The overlap between CCA outliers, DEGs and genes contained within significant WGCNA modules was examined for each tissue independently. For each gene set, we identified the function by performing independent GO and KEGG analyses (Table S3). In the lung, there were 76 overlapping genes out of 12,667 with functions related to apoptosis, immune function, inflammation, angiogenesis, glutamate, membrane component, nervous system development, mitochondria and calcium (see Table S3 for the full list). In the liver, 34 genes overlapped out of 10,244 related to gluconeogenesis, immune system, insulin, etc. (Table S3). In the kidney, 17 genes overlapped out of 11,908 related to sodium, angiogenesis, apoptosis, gluconeogenesis, etc. (Table S3). In the hypothalamus, 46 genes overlapped out of 11,025 related to calcium, digestion and absorption, glutamate, immune system, mitochondria, nervous system development, etc. (Table S3). In the GI tract, 442 genes overlapped out of 12,200 related to cholesterol, digestion and absorption, glutamate, inflammation and insulin (Table S3).

## DISCUSSION

Currently, there is limited research on the effects of low-fat diet as studies have historically focused on the consequences of high-fat diets (Abdel-Rahman, 2010; Hino et al., 2017; Kohsaka et al., 2007; Leone et al., 2015; Song et al., 2004). Previous work has explored diet composition and variation (Bachmanov et al., 2002; Beale et al., 2018; De la Iglesia et al., 2016; Hochman and P. Kotler, 2006; Hope and Parmenter, 2007; Nagy, 1994). Here, we expand on these studies to characterize the physiological and genomic mechanisms in response to a low-fat diet. This is of particular interest as diet directly affects both energy and water homeostasis (Schmidt-Nielsen and Adolph, 1964). Consumers respond to diets with multiple organ systems and physiological processes, a difficult signal to identify as gene expression highlights differences between tissues rather than processes coordinated by two or more tissues. It has been demonstrated that distributional changes of plants and consumers, in response to a changing climate, is occurring (Moritz et al., 2008; Parmesan, 2006), resulting in diet shifts and unexpected organismal responses.

We investigated the impact of dietary fat in a desert-adapted rodent using a multi-tissue gene expression dataset with whole-organism physiological responses to explore the interplay between physiological and genomic responses in harsh habitats. We observed that mice fed a lower-fat diet had (1) differences in expression levels in immune system genes, (2) gene expression changes in circadian regulating genes and (3) expression changes related to mitochondrial function. In regions characterized by severe water scarcity, even subtle deviations of overall organismal immune function and water balance may significantly affect physical performance and consequently survival and reproductive success. Our findings contribute to a better understanding of the organismal significance of food availability and dietary preferences and suggest that survival might be less dependent on a high-fat diet when water is accessible.

### Diet and dehydration

We previously (Blumstein et al., 2024) observed that mice on a diet lower in fat had a pronounced increase in water loss compared with those on a higher-fat diet. While animals had *ad lib* access to water in this experiment, the differences in water loss may be important in arid environments where water scarcity is extreme. Even subtle differences in water balance could profoundly impact cognitive function, physical performance and, consequently, survival and reproductive success (Cunningham et al., 2021; Riebl and Davy, 2013). Key electrolytes sensitive to hydration status, such as serum Na and osmolality, and Hct were elevated in animals fed the LF diet, indicating a difference in water balance. However, synthetic markers of pathological renal impairment, BUN and Cr, did not significantly differ between treatments (further discussed in Blumstein et al., 2024). In the study described here, we found several significant genes identified in our consensus gene sets related to the management of Na [*SLC9A5* (lung), *TNR* (hypothalamus), *SLC38A4* and *SLC17A1* (kidney); Table S3] and osmolality [*ADCYAP1R1* and *PKLR* (hypothalamus), *ITLN1* (lung), *SLC2A9* (liver), *GJB1* and *PPP1R3G* (kidney); Table S3], suggesting that varied fat content results in a solute concentration response. However, genetic markers of renal impairment (see MacManes, 2017) were not identified in the consensus gene set (Table S3).

During dehydration, the renin–angiotensin–aldosterone system (RAAS) and vasopressin are upregulated for solute management (Aisenbrey et al., 1981; Bouby and Fernandes, 2003; Greenleaf, 1992; Roberts et al., 2011). Water-deprived cactus mice have a consistent and robust whole-body RAAS response for water balance regulation (Blumstein and MacManes, 2024). In the current experiment, the primary genes responsible for this response, *AGT* (angiotensinogen), *ACE2* (angiotensin-converting enzyme), *REN1* (renin), *CMA1* (converts angiotensin I to angiotensin II) and *AGTR1* (angiotensin II receptor), were either not found in our datasets (*CMA1* and *REN1*) or downregulated in LF diet mice [*AGT* and *AGTR1* (GI tract)], which is contrary to our prediction. Only *ACE2* and *AGTR1* (lung) were found to be upregulated in LF diet mice. Additionally, the vasopressin pathway (hsa04962; Kanehisa et al., 2023) is only partially differentially expressed, with *RAB5C*, *CREB3L2* and *AQP3* significantly upregulated while *AVPI1*, *RAB11B* and *VAMP2* were significantly downregulated in LF diet mice in the GI tract. *DYNLL2* was significantly upregulated and *VAMP2* was significantly downregulated in LF diet mice in the lung. Lastly, *AVP* was significantly upregulated in LF diet mice in the hypothalamus. These responses are very different to those seen in Blumstein and MacManes (2024), where the RAAS pathway was found to be activated, and suggest that mice were not dehydrated to the levels that would stimulate a systematic and whole-organismal response.

The management of the consequences of altered fluid balance during dehydration involves processes such as angiogenesis or vascular remodeling that maintain perfusion, addressing challenges from reduced fluid availability and potential hemoconcentration (Blumstein and MacManes, 2024). In the study described here, we found two upregulated genes identified in our consensus gene sets, *ANG* (lung) and *ADTRP* (liver and kidney) involved in angiogenesis (Table S3). However, a WGCNA module in the kidney grouping into the GO term blood vessel development [GO:0001568] was downregulated. Furthermore, genes related to coagulation factors during dehydration that mitigate risks associated with increased blood viscosity and clot formation were downregulated. We found one downregulated module in the hypothalamus relating WLR to coagulation [coagulation (GO:0050817) and blood coagulation, fibrin clot formation (GO:0072378)] as well as two downregulated genes identified in our consensus gene sets, *F5* and *C8G* (GI tract) (Table S3) related to coagulation. However, we also found three genes upregulated in the GI tract related to coagulation (*CFI*, *PLG* and *F13B*; Table S3). This suggests that the response to dehydration on a LF diet is not a global one, unlike what is discussed in Blumstein and MacManes (2024), because the mice had water *ad libitum* and were not dehydrated.

Lastly, in our previous studies (Blumstein and MacManes (2023, 2024), we hypothesized that water-deprived mice reduce the amount of solid food intake in a response known as dehydration-associated anorexia. This response reduces the amount of water required for digestion and allows for water reabsorption from the kidneys and GI tract back into systemic circulation (Rowland, 2007; Watts and Boyle, 2010). However, unlike those studies, we did not observe that the GI tract was empty of food and feces in the mice fed the LF diet, suggesting that mice are not limiting food intake. If food intake was restricted, we would expect to see genes involved in glycogenolysis and gluconeogenesis (Salter and Watts, 2003; Schoorlemmer and Evered, 2002; Watts and Boyle, 2010). During fasting or starvation, pathways are designed to maintain blood glucose levels. However, in the current study, ample food and water were supplied but glycogenolysis and gluconeogenesis were significantly different when comparing males and females separately for the SD versus the LF diet treatments (Table 1). More specifically, several genes from our consensus results (Table S3) were significantly upregulated in LF diet mice for gluconeogenesis [*PKLR* (hypothalamus), *ADH4*, *PKLR*, *HKDC1*, *ALDOB*, *PCK1*, *FBP1* (GI tract), *PFKFB1* (liver; however, this gene inhibits gluconeogenesis); Table S3] and several were also significantly downregulated in LF diet mice for gluconeogenesis [*CYP2E1* (liver), *PPP1R3G* (kidney), *ADH4*, *ENO2* (GI tract); Table S3]. It is important to note that the two diets differ in the amount of glucose, so understanding whether glucose levels and gluconeogenesis are different as a result of diet or dehydration is not currently possible. Further studies should measure food and water intake directly to further explore potential mechanism for the observed differences.

### Diet and the mitochondria

The complex interplay between diet, mitochondrial function and metabolic homeostasis underscores the dynamic nature of cellular physiology (Dimitriadis et al., 2011). The processing of glucose and fatty acids is controlled via reciprocal regulation between fatty acid oxidation and glucose metabolism, wherein increased glucose consumption suppresses fatty acid oxidation and vice versa (Hue and Taegtmeyer, 2009). The mitochondria play pivotal roles in these regulatory processes, contributing to overall metabolic homeostasis (Caton et al., 2011; Saltiel and Kahn, 2001). When fatty acid oxidation is prioritized, there is an increase in acetyl-CoA and reduced nicotinamide adenine dinucleotide (NADH) levels, which negatively regulates the catalytic activity of PDH through allosteric inhibition and the activation of PDK. During fasting periods, the expression of the *PDK* gene is upregulated by fatty acid-induced peroxisome proliferator-activated receptor (PPAR) signaling (Sugden and Holness, 2006). This regulatory mechanism creates a positive feedback loop that promotes fatty acid oxidation during nutrient scarcity, preserving glucose for essential biosynthetic processes and brain metabolism. Hormones such as glucagon-like peptide-1 (GLP-1) and bile acids modulate glucose metabolism and insulin secretion, highlighting the intricate hormonal regulation of metabolic pathways (Jin and Weng, 2016; Kumar et al., 2012; Thomas et al., 2009; van Nierop et al., 2017; Vettorazzi et al., 2016). In this experiment, we saw elevated fatty acid oxidation with the SD, and glucose metabolism with the LF diet in mitochondrial genes; however, we also found results contradicting this, as discussed below.

In the current study, there was a robust mitochondrial signal. In the kidney, liver, lung and hypothalamus, several GO terms related to the mitochondria were significantly upregulated for mice fed the LF diet. Additionally, we found many overlapping GO terms related to the mitochondria from the independent WGCNA sex and diet modules in the liver and kidney [inner mitochondrial membrane protein complex (GO:0098800), mitochondrial envelope (GO:0005740), mitochondrial matrix (GO:0005759), mitochondrial inner membrane (GO:0005743), mitochondrial membrane (GO:0031966), mitochondrial translation (GO:0032543), mitochondrion (GO:0005739) and mitochondrial protein-containing complex (GO:0098798); Fig. 2] and one GO term in the WLR module for the GI tract [mitochondrial translation (GO:0032543)]. Additionally, *PDK1* and *PDHA1*, convergent genes in cellular energy metabolism (described above) were both significantly differentially expressed in the GI tract, with *PDHA1* being upregulated in mice fed the LF diet and *PDK1* upregulated in mice fed the SD. Further, 202 genes were significantly downregulated in the LF treatment in the GI tract and were grouped into the GO term cellular response to insulin stimulus (GO:0032869), as well as nine genes from the consensus GI tract list (*ADCY2*, *ATP1A2*, *HKDC1*, *KCNMA1*, *PPP1R3B*, *PRKCB*, *RIMS2*, *RYR2*, *SNAP25*; Table S3) and three genes from the consensus liver list (*HNF4A*, *IGFALS*, *ITIH2*; Table S3) related to insulin were downregulated, suggesting that the mitochondria are successfully contributing to overall metabolic homeostasis, further explaining the insignificant differences in EE reported in Blumstein et al. (2024).

### Diet, the immune system and the microbiome

The significant increase in water loss and elevated levels of key electrolytes sensitive to hydration status, including serum Na, osmolality and Hct, in mice on the LF diet compared with those on a higher-fat diet indicates a distinct difference in water balance between the two diets (Table 1). As water levels decrease, essential functions are affected, including circulation (Fountain et al., 2023; Georgescu et al., 2017; Santos et al., 2019), temperature regulation (Boonstra, 2004; Canale et al., 2012; Dammhahn et al., 2017; Wingfield et al., 1992) and nutrient transport (Alim et al., 2019; Alonso et al., 2005). Dehydration-induced stress can activate a stress response and suppress immune system function, impairing the body's ability to fight off infections and pathogens (Mitchell et al., 2002; Sies, 2015).

Further, diet plays a dual role as both a fuel source and modulator of both the GI tract and the microbiome (Henderson et al., 2015; Keeney and Finlay, 2011; Wu et al., 2011). Fatty acids enhance gene expression in innate immune cells (Holzer et al., 2011; Wong et al., 2009; Zeng et al., 2016; Zhao et al., 2007). We observed several genes in our consensus gene lists related to immune response upregulation in mice fed a SD [*CNTN2*, *NFASC*, *CD1D*, *CCL11* (lung), *A1BG*, *C8A*, *C9*, *ITIH4*, *PCDHGB1*, *ULBP1* (liver), *PTPRZ* (hypothalamus), *PIGR* (kidney), *IL7R* (GI tract); Table S3] and several genes related to inflammation upregulated in mice fed a SD [*ITIH1*, *CCL11*, (lung) and *ITIH1* (liver), and *ADCY8*, *ASIC4*, *PRKCB* (GI tract); Table S3]. However, it is important to note that inflammation-related genes were only identified in three of the five tissues, suggesting an isolated response to altered fat content.

Beyond their inflammatory effects, fatty acids influence bacterial survival and proliferation in the gut (Zeng et al., 2016), partly by disrupting microbial cell membranes (Chen et al., 2011). Consequently, dietary lipid composition can impact gut microbiota colonization and diversity (Jumpertz et al., 2011; Wu et al., 2011), and changes in gut bacteria composition exert ongoing selective pressure on immune modulation by fat (Hooper et al., 2012; Karlsson et al., 2013). The GI tract, lung and liver all had a significant WGCNA module with downregulated genes in the LF treatment that grouped into GO terms related to a defense response and or a response to other organisms [regulation of defense response (GO:0031347), response to other organism (GO:0098542), defense response to virus (GO:0051607), response to bacterium (GO:0009617), defense response to symbiont (GO:0140546); Fig. 2]. An unsuccessful response to infections has been shown to alter behavior in other studies (Cavadini et al., 2007), resulting in the downregulation of clock gene expression and disruption of the circadian clock, leading to a reduction in the amplitude of circadian rhythms (discussed below). Future studies should explore shifts in gut microbiome community structure.

### Diet and circadian rhythm

Circadian rhythmicity of metabolism profoundly influence organ function and systemic homeostasis in mammals, with the most robust circadian cues being light and food (Pittendrigh 1960; Roenneberg and Merrow 2005). The circadian regulation of physiological processes involves intricate interactions between the central clock in the hypothalamus and peripheral clocks in various organs (Hirota and Fukada, 2004; Xie et al., 2019). While light controls the central clock, peripheral clocks are influenced by rhythmic feeding and fasting cycles, nutrient levels, energy availability, redox levels and diet composition (Kohsaka et al., 2007; Leone et al., 2015; Tahara et al., 2018; Voigt et al., 2014; Zarrinpar et al., 2014). Metabolic parameters play crucial roles in feedback regulation, affecting core function (Kohsaka et al., 2007; Leone et al., 2015; Zarrinpar et al., 2014). Lipid absorption in the small intestine, regulated by clock genes, displays diurnal variations affected by light and feeding cues (Pan and Hussain, 2009). In our consensus gene sets (Table S3), we observed several genes whose primary function delt with digestion and absorption. Interestingly, genes in the consensus GI tract list related to fat digestion and absorption were upregulated in mice fed the LF diet (*FABP1*, *PNLIPRP2*, *ABCG8*, *FABP2*, *ABCG5*, *PLA2G12B*, *NPC1L1*, *MTTP*, *APOA4*, *APOB*; Table S3) while genes in the consensus GI tract list related to carbohydrate digestion and absorption were upregulated in mice fed the SD (*PRKCB*, *HKDC1*, *SLC2A2*, *ATP1A2*, *PDHA1*; Table S3).

The kidney, liver and GI tract exhibit rhythmic regulation of blood flow, urine production and metabolic pathways, modulating processes such as detoxification, glucose homeostasis and lipid metabolism (Solocinski and Gumz, 2015; Zwighaft et al., 2016). Also, gluconeogenesis and glycogen synthesis are under circadian control, involving interactions between core clock components and metabolic regulators (Lamia et al., 2008). Mitochondrial dynamics and function also display circadian rhythmicity, regulated by clock genes (Jacobi et al., 2015; Kohsaka et al., 2014; Magnone et al., 2015) and influenced by diet composition and feeding time (Neufeld-Cohen et al., 2016). Lipids, essential for mitochondrial membrane integrity and energy production, exhibit circadian accumulation patterns (Aviram et al., 2016), synchronized with the molecular clock and feeding cues (Osman et al., 2011).

We found several overlapping GO terms related to circadian rhythm from the independent WGCNA analysis in the kidney, liver and lung [circadian rhythm (GO:0007623), entrainment of circadian clock by photoperiod (GO:0043153), photoperiodism (GO:0009648), positive regulation of response to stimulus (GO:0048584) and regulation of circadian rhythm (GO:0042752), circadian regulation of gene expression (GO:0032922), circadian behavior (GO:0048512), circadian regulation of translation (GO:0097167), entrainment of circadian clock (GO:0009649), entrainment of circadian clock by photoperiod (GO:0043153); Fig. 2]. Further, several genes in the consensus GI tract list related to circadian rhythm were downregulated in mice fed the LF diet (*GNAO1*, *RYR2*, *GNG3*, *PRKCB*, *ADCY2*, *CACNA1G*, *GRIN1*; Table S3). It is important to note that the core clock genes in the KEGG pathway circadian rhythm (hsa04710; Kanehisa et al., 2023) *CLOCK*, *ARNTL* (coded as *BMAL1*), *PER*, *CRY*, *ROR* and *REV-ERB* were not significant differentially expressed in most tissues, with the exception of *CRY1* and *PER1* in the GI tract and *ARNTL* and *CRY1* in the lung. Nevertheless, many of these genes were present in significant WGCNA modules and while not significantly differentially expressed, they were downregulated in the LF diet compared with the SD. This could suggest these genes may have small changes in gene expression but big effects or that other genes that display circadian rhythmicity are driving the expression patterns in the WGCNA analysis (Fig. 2).

### Conclusion

We have identified several responses employed by the cactus mouse to altered dietary fat content, providing insights into physiological adaptations to increasingly water-stressed plant-based food resources as a consequence of climate change. We describe (1) gene expression changes in immune system genes, (2) gene expression changes in circadian regulating genes and (3) expression changes related to mitochondrial function when comparing cactus mice fed a LF diet with those fed a SD. Our results suggest that a LF diet could limit the capacity of desert animals to tolerate reduced access to free water, as is common in arid environments, as well as paving the way for further exploration and a holistic comprehension of the impact of diet on survival strategies in harsh habitats and the potential implications for long-term population viability amid changing climates.

The interplay between metabolism, diet, the immune response and circadian rhythms is complex and multifaceted, with implications for overall health and disease. Understanding these interactions sheds light on the intricate regulatory mechanisms that govern physiological processes in a time-dependent manner. There is a push to incorporate -omics with physiology to further understand these processes but currently there are challenges. The analysis discussed is a single contemporary ‘snapshot’ of gene expression. This leaves a mismatch in time scales for contemporary studies as the desired physiological datasets are continuous and contain temporal patterns while gene expression studies that include temporal variation may quickly become overwhelming in cost, scale and analytical complexity. While we were able to identify genes and GO terms related to circadian rhythm, we do not yet have data to confirm this result and future studies should explore this. Additionally, grouping genes by function for comparison between organ systems is further complicated because of the hierarchical nature of GO terms. Functional overlap could be obscured by a parent term being assigned to one tissue while a child term is assigned to another. Lastly, we were able to identify gene expression differences in immune system genes for mice on the different diets and a very large change in gene expression in the GI tract. We hypothesize that the microbiome is playing a role in this response and encourage further studies to characterize differences in the microbiome. Further knowledge of the underpinnings of the physiological phenotypes will be instrumental in discovering more ​​mechanistic links. This will enable a shift from describing correlation to identifying causation of the diverse phenotypes that contribute to phenotypic variation and a rapid response to environmental change, and combining techniques may allow for a more complete picture of ecological interactions and evolutionary processes.

Future research should focus on a comprehensive analysis of the effects of all dietary macronutrients – fats, carbohydrates and proteins – systematically measuring food and water intake for each diet, thereby disentangling their individual contributions and their interplay with energy and water balance. Additionally, the experiment discussed here is limited to gene expression data collected at noon. By truncating the physiology data to the mean of the data in the last hour of the experiment, we were able to match that time of the gene expression data. However, previous studies have shown that both physiological measurements (Blumstein and MacManes, 2023; Blumstein et al., 2024; Colella et al., 2021b) and gene expression (Deota et al., 2023; Kumar Jha et al., 2015; Schibler, 2007) are cyclical and even ‘habitat’ dependent. Expanding future studies to include gene expression at various time points could lead to a deeper understanding of how dietary composition and timing influence health and behavior.

## Supplementary Material

10.1242/jexbio.247978_sup1 Supplementary information

Table S1. Mapping rates for all samples sequences.

Table S2. Gene ontology terms for each DESeq comparison (all mice the standard diet to all mice on the low-fat diet, males versus females on the standard diet, and males versus females on the low-fat diet) and each WGCNA analysis in the lung, liver, gastrointestinal tract, hypothalamus, and kidney of Peromyscus eremicus.

Table S3. Genes identified in all three analyses (i.e. significantly differentially expressed genes, assigned to a significant module in WGCNA, and are outliers in CCA) for the lung (lu), liver (liv), hypothalamus (hyp), kidney (kid), and gastrointestinal tract (gi) grouped by the general function of the gene based on GO analysis and KEGG analysis.
