# Supplementary material for: Impacts of dietary fat on multi tissue gene expression in the desert-adapted cactus mouse
Source: J Exp Biol. 2024 Dec 16;227(24):jeb247978. doi: 10.1242/jeb.247978 (PMC11698062; doi:10.1242/jeb.247978)
Supplement: Supplementary information [file jexbio-227-247978-s1.pdf]

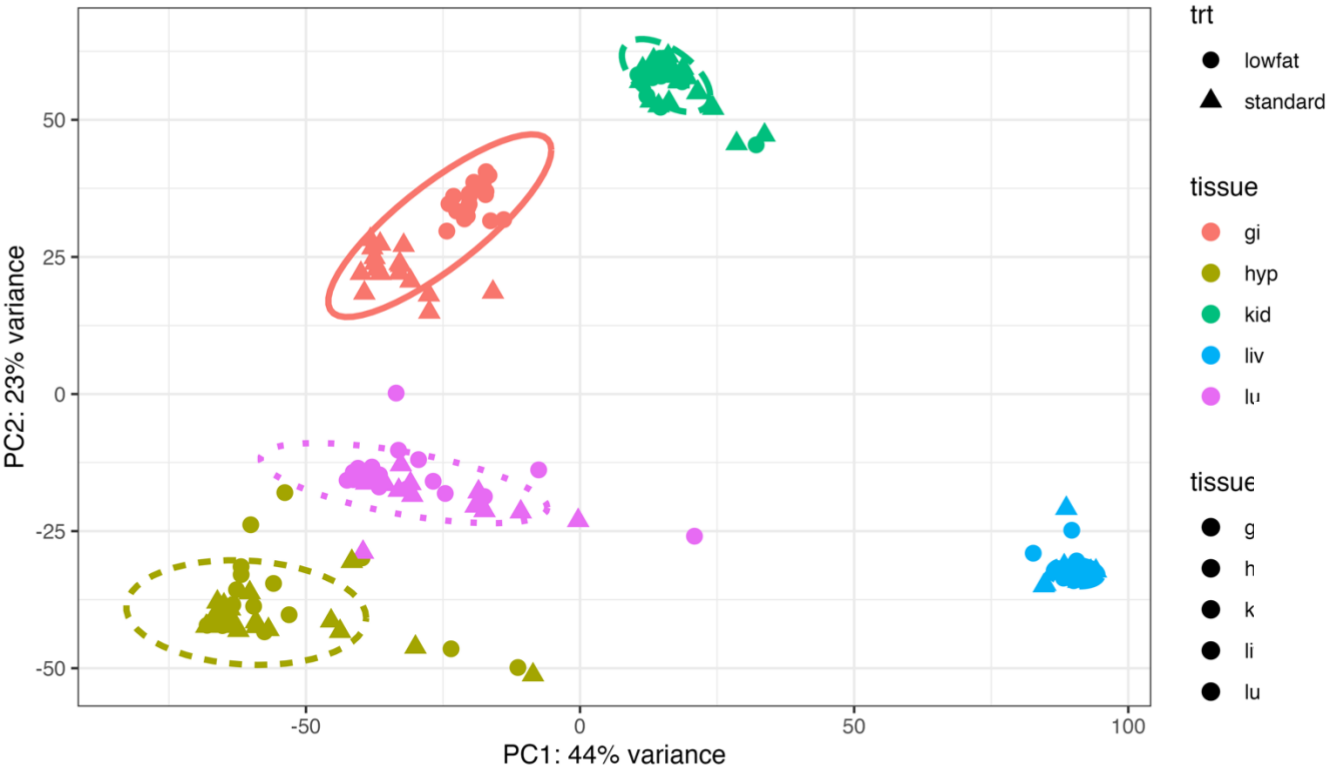

Fig. S1. Principal component analysis of gene expression of the lung (lu), liver (liv), gastrointestinal tract (gi), hypothalamus (hyp), and kidney (kid) of *Peromyscus eremicus*. The axes are labelled with the proportion of the data explained by principal components 1 and 2.

Table S1. Mapping rates for all samples sequences.

Available for download at
https://journals.biologists.com/jeb/article-lookup/doi/10.1242/jeb.247978#supplementary-data

Table S2. Gene ontology terms for each DESeq comparison (all mice the standard diet to all mice on the low-fat diet, males versus females on the standard diet, and males versus females on the low-fat diet) and each WGCNA analysis in the lung, liver, gastrointestinal tract, hypothalamus, and kidney of Peromyscus eremicus.

Available for download at
https://journals.biologists.com/jeb/article-lookup/doi/10.1242/jeb.247978#supplementary-data

Table S3. Genes identified in all three analyses (i.e. significantly differentially expressed genes, assigned to a significant module in WGCNA, and are outliers in CCA) for the lung (lu), liver (liv), hypothalamus (hyp), kidney (kid), and gastrointestinal tract (gi) grouped by the general function of the gene based on GO analysis and KEGG analysis.

Available for download at
https://journals.biologists.com/jeb/article-lookup/doi/10.1242/jeb.247978#supplementary-data
